# Diagnostic challenges and management strategies of pulmonary mucosa-associated lymphoid tissue lymphoma: a case report and literature review

**DOI:** 10.3389/fmed.2025.1719542

**Published:** 2025-12-18

**Authors:** Jian Tang, Bijun Yang, Yang Bai

**Affiliations:** 1 Department of Respiratory and Critical Care Medicine, The People's Hospital of Neijiang Dongxing District, Neijiang, China; 2 Department of Respiratory and Critical Care Medicine, The First Affiliated Hospital of Chongqing Medical University, Chongqing, China

**Keywords:** endobronchial ultrasound, extranodal marginal zone lymphoma, interventional pulmonology, mucosa-associated lymphoid tissue, postgraduate education, transbronchial cryobiopsy

## Abstract

**Background:**

Pulmonary mucosa-associated lymphoid tissue (MALT) lymphoma is a rare extranodal B-cell lymphoma, representing the most common primary pulmonary lymphoma (< 0.5% of all lung malignancies). Diagnosis is difficult because of its nonspecific clinical and radiological characteristics, which often resemble infections, inflammatory diseases, or other malignancies. Typical imaging findings include nodules, consolidations, or, less frequently, interstitial patterns such as ground-glass opacities. A conclusive diagnosis requires histological examination, augmented by immunohistochemistry and molecular investigations, to confirm clonality. Conventional biopsies may yield inconclusive outcomes due to small sample size and delicate cytologic atypia.

**Case presentation:**

A 36-year-old non-smoking Asian female with no history of pulmonary tuberculosis, malignancies, or autoimmune disorders was incidentally found to have patchy opacities in the left lower lobe on routine chest radiography in January 2024. She remained asymptomatic over the 18-month disease progression period, lacking respiratory or systemic symptoms, whereas follow-up imaging indicated progression to bilateral, multifocal flocculent opacities. Initial investigations, including transbronchial forceps biopsy and bronchoalveolar lavage fluid analysis through next-generation sequencing, reported only non-specific chronic inflammation and no pathogens. Empirical treatment with moxifloxacin (400 mg daily for 14 days), targeting common pathogens of community-acquired pneumonia, yielded no improvement. A definitive diagnosis was achieved by endobronchial ultrasound-guided transbronchial cryobiopsy (EBUS-TBCB), which produced larger, well-preserved tissue specimens. Histopathological and immunohistochemical examination indicated a dense, angiocentric infiltration of CD20-positive B cells, accompanied by monoclonal rearrangement of immunoglobulin genes, confirming extranodal marginal zone lymphoma of the MALT type. Staging with ^68^Ga-CXCR4 positron emission tomography (PET)/computed tomography (CT) demonstrated hypermetabolism in bilateral pulmonary opacities and multiple nodal stations, indicative of stage IV disease. Considering the asymptomatic status and excellent performance status, active surveillance was recommended in this patient.

**Conclusion:**

This case illustrates the important role of advanced biopsy techniques, such as EBUS-TBCB, in acquiring sufficient tissue for diagnosing pulmonary MALT lymphoma when conventional methods fail. It further underscores the ^68^Ga-CXCR4-targeted PET/CT for precise staging. Despite the advanced stage, the indolent nature of MALT lymphoma often allows for a plan of active surveillance in selected asymptomatic patients, emphasizing the crucial role of a multidisciplinary, risk-adapted strategy.

## Introduction

Pulmonary mucosa-associated lymphoid tissue (MALT) lymphoma, an extranodal marginal zone B-cell lymphoma originating from bronchus-associated lymphoid tissue, is the predominant subtype of primary pulmonary lymphoma (1–3). This rare disease constitutes around 70 to 80% of all primary pulmonary lymphomas; however it represents less than 0.5% of primary lung cancers and approximately 0.4% of all non-Hodgkin lymphomas (4). The clinical presentation of pulmonary MALT lymphoma is often nonspecific and heterogeneous. Patients may have nonspecific respiratory symptoms (such as dry cough and mild dyspnea), low-grade systemic “B symptoms” (fever, night sweats, or weight loss), or may be asymptomatic. This heterogeneity frequently leads to diagnostic delays or initial misdiagnosis, with a considerable proportion of cases (up to one-third) being diagnosed inadvertently during imaging performed for unrelated reasons (2, 5). Computed tomography (CT) has greater sensitivity than standard radiography in delineating pulmonary MALT lymphoma, which often presents with bilateral and multifocal distribution without specific topographic predominance (6, 7). The most frequent morphological patterns seen on CT include consolidations, nodules, or masses, with airways visible within the lesions (air bronchograms) (6, 7). Occasionally, an interstitial pattern characterized by micronodules, ground-glass opacities, and septal thickening may be seen (8, 9). The definitive diagnosis of pulmonary MALT lymphoma requires histological examination of biopsy-derived tissue specimens, integrating morphological evaluation with immunophenotypic profiling and molecular analysis (10).

This case report details a 36-year-old non-smoking female diagnosed with stage IV pulmonary MALT lymphoma after endobronchial ultrasound-guided transbronchial cryobiopsy (EBUS-TBCB). This case exemplifies the diagnostic challenges associated with this rare disease and highlights the evolving role of advanced bronchoscopic techniques in obtaining a conclusive diagnosis.

## Case presentation

**Figure 1:**
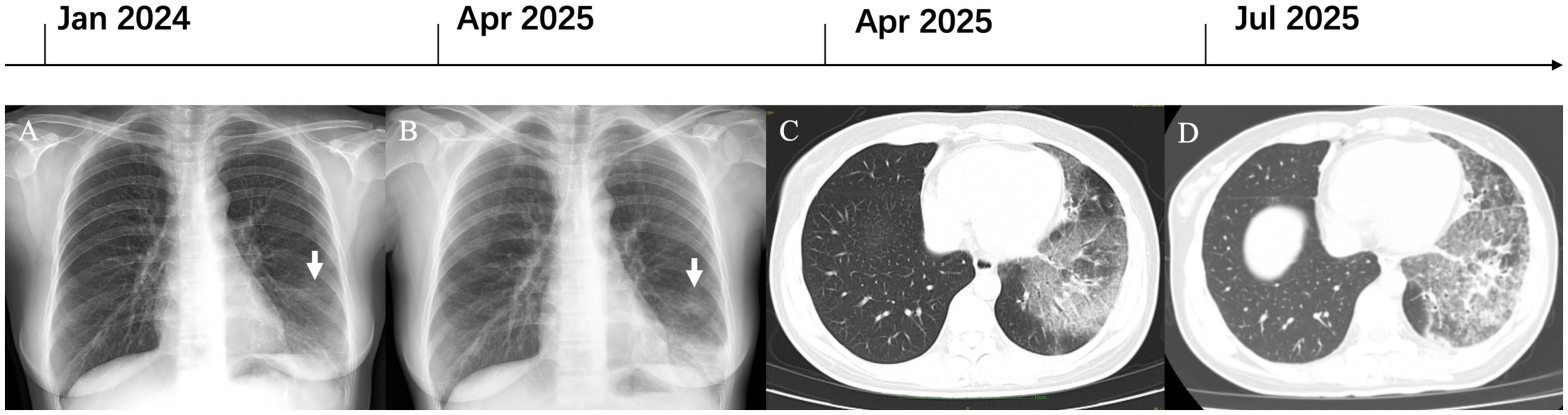
Imaging reveals progressive lung opacities in pulmonary MALT lymphoma. **(A)** Patchy opacification confined to the left lower lobe (white arrow, Jan 2024). **(B)** Progression to consolidation with patchy infiltrates (white arrow, Apr 2025). **(C)** Multifocal flocculent opacities with bronchovascular markings on the contrast CT scan, predominantly in the left lower lobe. **(D)** Persistent multifocal patchy and flocculent opacities, with new subtle interlobular septal thickening (July 2025). MALT, mucosa-associated lymphoid tissue.

A 36-year-old life-long non-smoking Asian female, with no prior history of pulmonary tuberculosis, malignancies, or autoimmune disorders, underwent a routine chest radiograph in January 2024, which revealed patchy opacification in the left lower lobe (Figure 1A). She remained asymptomatic and received no therapy. Follow-up radiography in April 2025 revealed progression to consolidation and patchy infiltrates localized to the same lobe (Figure 1B). Subsequent contrast-enhanced CT demonstrated bilateral multifocal flocculent opacities with bronchovascular markings, mostly in the left lower lobe (Figure 1C). Diagnostic bronchoscopy revealed no structural abnormalities, and transbronchial forceps biopsies indicated only non-specific chronic inflammation. Next-generation sequencing of bronchoalveolar lavage (BAL) fluid detected no bacterial, mycobacterial, viral, or fungal pathogens. Empirical treatment with moxifloxacin (400 mg daily for 14 days), targeting common pathogens of community-acquired pneumonia, produced no radiological improvement. Repeat CT in July 2025 illustrated persistent multifocal patchy and flocculent opacities with subtle interlobular septal thickening (Figure 1D), with no mediastinal or hilar lymphadenopathy. The patient was therefore referred to our tertiary center for further evaluation.

Upon admission, the patient reported no cough, sputum production, dyspnea, hemoptysis, fever, or weight loss. Vital signs were normal as follows: body temperature 36.5 °C, respiratory rate 16 breaths per minute, oxygen saturation 98% (on room air), blood pressure 128/72 mmHg, heart rate 70 beats per minute. Chest auscultation revealed diminished breath sounds over the left lower lobe without crackles or wheezes. Extensive infectious and autoimmune panels (including HIV, interferon-gamma release assay, anti-neutrophil cytoplasmic antibodies, connective-tissue-disease screen) were negative.

**Figure 2:**
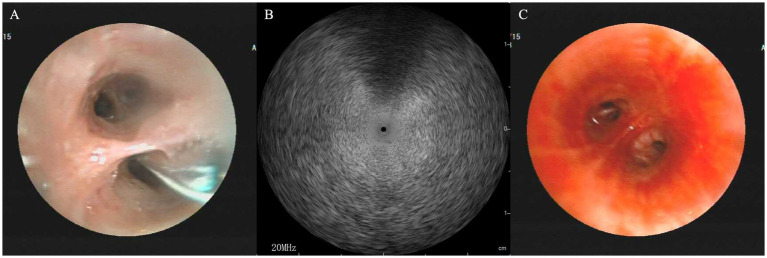
EBUS-TBCB procedure. **(A)** Pre-procedural radial ultrasound mapping of the left lower lobe basal segment. **(B)** Identification of a “blizzard sign” on EBUS imaging, confirming dense parenchymal infiltration. **(C)** Successful retrieval of four tissue specimens via cryoprobe (1.7 mm) without major bleeding. EBUS-TBCB, endobronchial ultrasound-guided transbronchial cryobiopsy.

**Figure 3:**
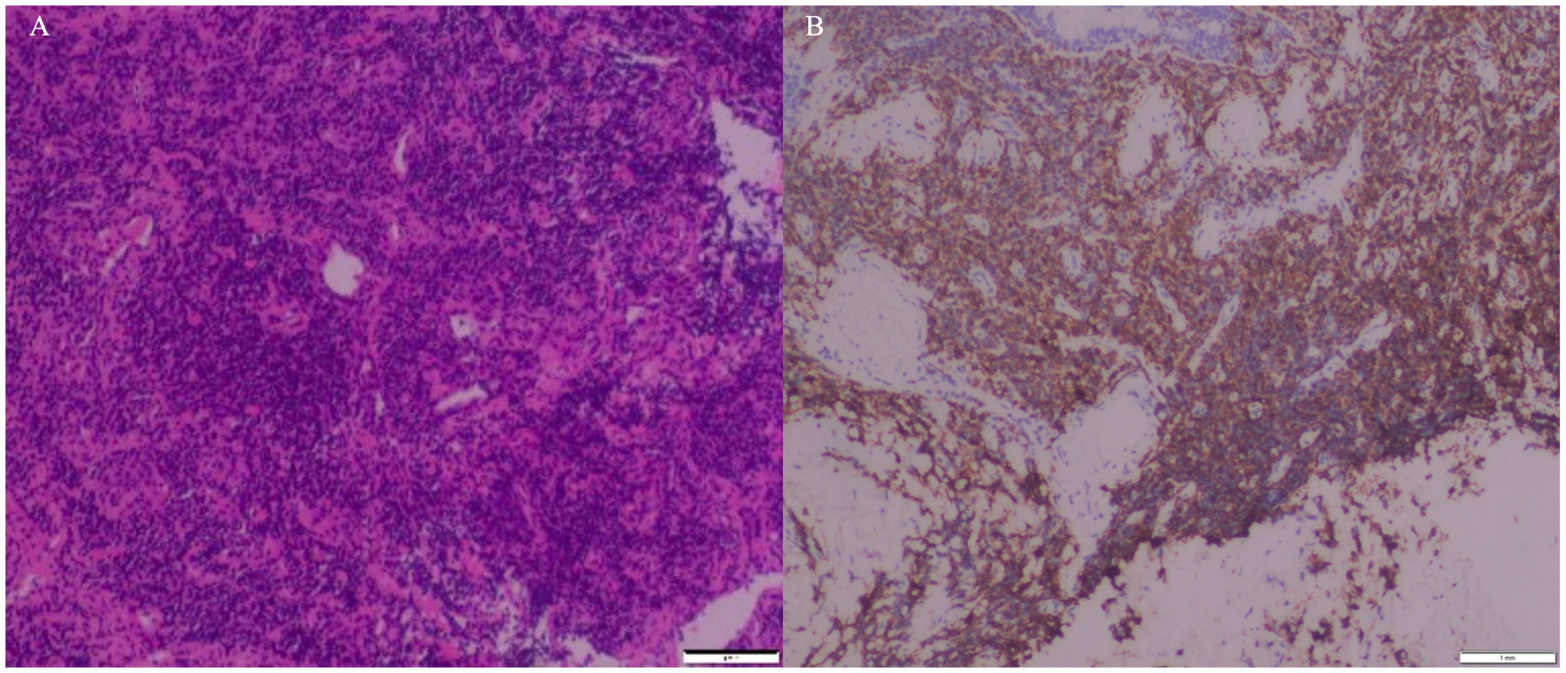
Pathology and IHC analysis of cryobiopsy confirm MALT lymphoma. **(A)** H&E staining: dense angiocentric atypical lymphocytes (×10). **(B)** IHC: strong CD20 membrane positivity (brown stain) in tumor cells, confirming B-cell origin (×10). H&E, Hematoxylin and Eosin; IHC, immunohistochemistry; MALT, mucosa-associated lymphoid tissue.

Considering the lack of response to antibiotic therapy and inconclusive initial investigations (including transbronchial biopsy and BAL next-generation sequencing), a more robust diagnostic modality was required. With the patient’s consent, we performed an endobronchial ultrasound-guided transbronchial cryobiopsy (EBUS-TBCB) under general anesthesia. A rigid bronchoscope (DUNBO, China) with jet ventilation (Carl Reiner GmbH, Vienna, Austria) was inserted to secure the trachea, assisted by a flexible bronchoscope (Fujifilm, Japan). A radial ultrasound probe (InnerMed, China) initially mapped the basal segments of the left lower lobe (Figure 2A), revealing “blizzard signs” (Figure 2B) suggestive of dense infiltration. A 1.7 mm cryoprobe (Erbe, Germany) was advanced through the working channel of the flexible bronchoscope; two 7-s freeze cycles were performed in both the anterior and lateral segments, obtaining four specimens (Figure 2C) without significant bleeding or pneumothorax. Pre-biopsy BAL differential showed 60% lymphocytes and < 1% eosinophils. Histopathological examination of the cryobiopsy specimens revealed dense, angiocentric infiltration of atypical lymphoid cells (Figure 3A). Immunohistochemistry analysis demonstrated strong membranous CD20 positivity (Figure 3B) with partial expression of CD3, CD5, CD10, Bcl-2, and Bcl-6; Cyclin D1 was negative. CD21/CD23 highlighted an intact follicular dendritic-cell meshwork, and the Ki-67 proliferation index was 20%. Cytokeratin, chromogranin, TTF-1, and napsin A were negative, excluding carcinoma. Polymerase-chain-reaction analysis found monoclonal rearrangements of the immunoglobulin B-cell heavy-chain (IgH) and kappa light-chain (IgK) genes. Epstein–Barr virus-encoded RNA *in situ* hybridization was negative. These findings collectively confirmed the diagnosis of extranodal marginal zone lymphoma of MALT type.

**Figure 4:**
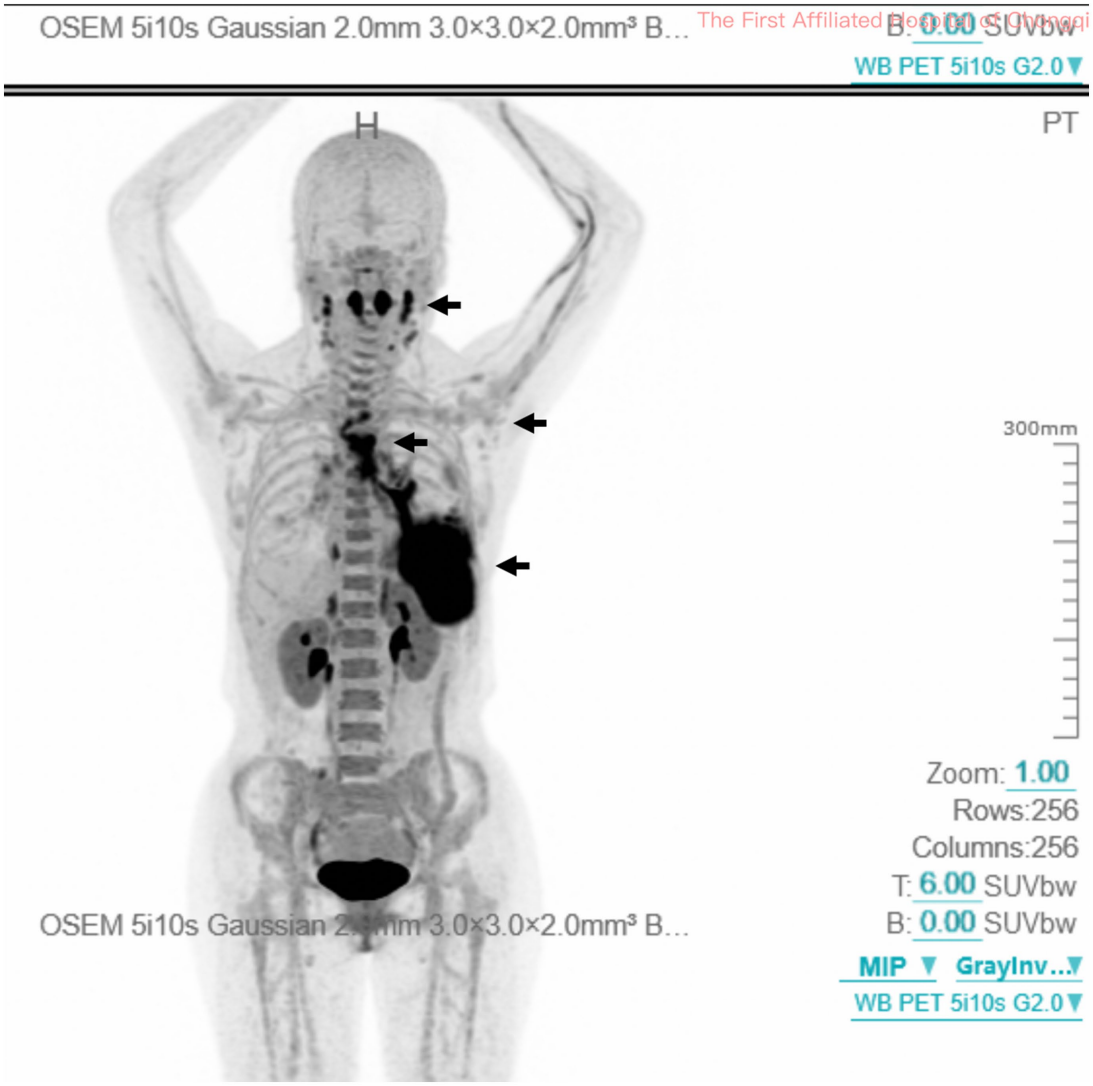
Whole-body ^68^Ga-CXCR4 PET/CT imaging for staging. Elevated uptake in bilateral pulmonary opacities, cervical lymph nodes, tonsillar tissue, and multiple intrathoracic nodal stations (black arrow). PET/CT, positron emission tomography/computed tomography.

Further evaluation with whole-body ^68^Ga-CXCR4 positron emission tomography/CT (PET/CT) identified elevated tracer uptake (SUVmax 11.3) in bilateral patchy opacities, most prominent in the left lower lobe. Hypermetabolism was observed in the bilateral cervical lymph nodes (SUVmax 10.6), tonsillar tissue (SUVmax 13.1), and intrathoracic lymph nodes, including bilateral hilar, mediastinal, and axillary stations (SUVmax 9), indicating malignant infiltration (black arrow, Figure 4). No other extranodal visceral involvement was identified. These imaging findings supported the diagnosis of stage IV extranodal marginal zone lymphoma of MALT type according to the Lugano classification (11). Given the patient’s complete absence of symptoms, excellent performance status (ECOG 0), and the indolent nature of MALT lymphoma, the multidisciplinary tumor board recommended active surveillance: clinical visits every 3 to 6 months for symptom review, physical examination, and hematology tests (complete blood count, metabolic panel, and lactate dehydrogenase), and chest CT if necessary to detect transformation or symptomatic progression. The patient continues to be followed per this plan and has remained clinically stable after 3 months of monitoring.

## Discussion

This case illustrates several critical elements in the diagnosis and management of pulmonary MALT lymphoma, emphasizing both the challenges and advancements in current practice. The initial presentation, asymptomatic with incidentally discovered radiographic opacities, corresponds with the recognized indolent nature of pulmonary MALT lymphoma, whereby up to one-third of patients are diagnosed inadvertently (1–5). The radiographic findings of bilateral, multifocal opacities coupled with septal thickening indicate a less common interstitial pattern, while the majority of patients usually manifest as nodular lesions or consolidations with air bronchograms (6–9). Such interstitial patterns appear in approximately 10% of cases, often resembling inflammatory or infectious diseases, thereby leading to diagnostic delays and initial misdiagnosis, as shown in this case (6, 8).

The diagnosis of pulmonary MALT lymphoma relies on tissue biopsy and an integration of morphological, immunophenotypic, and genetic criteria (5). In this case, conventional transbronchial forceps biopsies yielded only nonspecific chronic inflammation, highlighting the limitations of the small, fragmented specimens acquired by this method. TBCB can overcome these limitations by obtaining larger, structurally intact specimens compared to the conventional method (12, 13). EBUS-guided targeting of “blizzard signs” with TBCB achieved a diagnostic yield of 92.5% for diffuse parenchymal lung diseases (14). For rare conditions like pulmonary MALT lymphoma, EBUS-TBCB successfully diagnosed the disease after a failed traditional transbronchial lung biopsy, as the larger tissue samples enabled detection of lymphoepithelial lesions and light chain restriction (15, 16). The precise localization of lesions with EBUS can help avoid cryobiopsy-associated pneumothorax and major bleeding (17). Regarding chromosomal analysis for this patient, it was not performed primarily because the diagnosis of pulmonary MALT lymphoma had been sufficiently confirmed by the aforementioned comprehensive diagnostic workup including detailed histopathological examination (revealing dense angiocentric infiltration of atypical lymphoid cells), immunohistochemical profiling (e.g., strong membranous CD20 positivity, negative Cyclin D1, and intact follicular dendritic-cell meshwork via CD21/CD23), and molecular detection of monoclonal rearrangements of immunoglobulin genes (IgH and IgK) (10). Nevertheless, we fully recognize the significant clinical value of chromosomal analysis in MALT lymphoma. It serves a critical role in identifying specific chromosomal translocations (e.g., t(11;18)(q21;q21), t(1;14)(p22;q32), or t(14;18)(q32;q21)) that are closely linked to the pathogenesis, prognostic stratification, and even therapeutic decision-making for this disease (18).

^68^Ga-CXCR4 PET/CT is a sophisticated staging technique for MALT lymphoma, demonstrating efficacy in assessing bone marrow infiltration: sensitivity 85–90%, specificity 90–95%, positive predictive value 80–85%, negative predictive value 92–96% (19). The PET/CT scan in this patient revealed no bone marrow hypermetabolism, aligning with normal blood counts and eliminating the need for invasive bone marrow biopsy (11). The presence of bilateral pulmonary disease and nodal involvement across the diaphragm confirmed stage IV disease, emphasizing the importance of PET/CT in disease extent mapping. However, routine surveillance PET/CT scans for asymptomatic patients with pulmonary MALT lymphoma are not recommended because they carry a false-positive rate exceeding 20%, resulting in patient anxiety, radiation exposure, and unnecessary investigations (11).

Despite the advanced stage identified in this case, pulmonary MALT lymphoma typically follows an indolent course. The 5-year overall survival exceeds 85%, and many patients remain asymptomatic for extended periods (20). The patient’s young age, preserved performance status (ECOG 0), and low proliferative index (Ki-67 20%) are all favorable prognostic indicators (2, 21–23). The Lugano system facilitates risk-adapted treatment strategies, and for asymptomatic advanced-stage patients with low tumor burden, active surveillance is often the preferred approach, deferring systemic therapy until symptomatic progression or organ dysfunction occurs.

Notably, the patient’s disease progressed radiologically over 18 months without symptomatic worsening, highlighting the variable natural history of MALT lymphoma. While spontaneous regression has been rarely reported, progression to disseminated disease, as suggested by the ^68^Ga-CXCR4 PET-CT findings in this case, necessitates close observation or rituximab-based treatment (24). Rituximab-based regimens (e.g., R-CHOP, R-CVP, or bendamustine-rituximab) have proven effective, with high response rates and prolonged progression-free survival. Transformation to diffuse large B-cell lymphoma remains a recognized risk that warrants aggressive treatment strategies (25).

## Conclusion

This case underscores the diagnostic efficacy of EBUS-TBCB in acquiring sufficient tissue for integrating histologic, immunophenotypic, and molecular analyses, which are crucial for confirming pulmonary MALT lymphoma. The Lugano Classification, supported by advanced imaging modalities such as ^68^Ga-CXCR4 PET/CT, facilitates initial disease extent mapping. The indolent but progressive characteristics of this lymphoma necessitate prolonged CT monitoring to detect transformation or symptomatic progression, with rituximab-based treatments remaining effective upon need. Multidisciplinary evaluation and individualized management are paramount to optimizing outcomes in this rare entity.

## Data Availability

The original contributions presented in the study are included in the article/supplementary material, further inquiries can be directed to the corresponding author/s.
